# Narrative-Derived Indices of Metacognition among People with Schizophrenia: Associations with Self-Reported and Performance-Based Social Functioning

**DOI:** 10.3390/bs14040265

**Published:** 2024-03-23

**Authors:** Melissa F. V. Kilicoglu, Nancy B. Lundin, Kaley Angers, Aubrey M. Moe

**Affiliations:** 1 Department of Psychiatry and Behavioral Health, The Ohio State University, Columbus, OH 43210, USA; melissa.kilicoglu@osumc.edu (M.F.V.K.); nancy.lundin@osumc.edu (N.B.L.); 2 Department of Psychiatry, Neuropsychology Section, University of Michigan-Ann Arbor, Ann Arbor, MI 48109, USA; kangers@med.umich.edu

**Keywords:** language, psychosis, serious mental illness, recovery, narrative, social functioning, cognition

## Abstract

Metacognitive functioning—which broadly encompasses the mental processes involved in thinking about the thinking of one’s self and the thinking of others—is often impaired among individuals living with schizophrenia and may contribute to difficulties in social and interpersonal functioning. Although the majority of studies assessing metacognition among individuals with schizophrenia use standardized, laboratory-based measurements, an increasing number of studies have measured metacognitive capacity using natural language produced by individuals living with mental illness. At the same time, less is known about how language-derived indices of metacognitive function relate to key social outcomes among people with schizophrenia. The primary objective of this study was to employ a validated language coding system (the Metacognition Assessment Scale, Abbreviated; MAS-A) to assess metacognitive functioning from the spoken life narratives of individuals with schizophrenia (*n* = 32) and community controls (*n* = 15). Among individuals with schizophrenia, we also examined the associations between language-derived metacognition and measures of self-reported and performance-based social functioning. Our results suggest that most aspects of metacognition in our sample were not significantly diminished in people with schizophrenia compared to community controls. Unexpectedly, the MAS-A subscale related to one’s ability to master psychological difficulties was rated higher among individuals with schizophrenia. Further, our results suggest that among people with schizophrenia, higher metacognitive functioning in the domain of self-reflectivity was associated with poorer self-reported social functioning, while a greater metacognitive awareness of other individuals’ minds was associated with better scores on aspects of performance-based social functioning. Collectively, these results underscore the utility of assessing metacognitive functioning via life-story narratives to understand social outcomes and highlight possible aspects of resiliency among individuals who have experienced a serious mental illness.

## 1. Introduction

Schizophrenia is a serious and often disabling illness that impacts numerous individuals worldwide [1], with further reverberations on families, caregivers, and the overall societal burden and cost of illness [2,3,4]. Although schizophrenia is often associated with the hallmark “positive” symptoms of psychosis that are present during acute illness phases (e.g., hallucinations and delusions), many aspects of illness remain impaired even when positive symptoms remit, including a diminished quality of life, impaired social functioning, and altered self-perception [5,6]. Turner and Saeturn [7] found social satisfaction and connectedness to be more related to the perceived recovery from psychotic symptoms than other mental health symptoms (e.g., attentional difficulties and a depressed mood). Thus, clinicians and researchers have increasingly adopted phenomenological models of schizophrenia that emphasize affected individuals’ lived experience, including a focus on understanding the metacognitive underpinnings of schizophrenia.

Metacognition—broadly understood as the ability to think about one’s own thought process and that of others, as well as the ability to understand and respond to interpersonal challenges [8]—has been increasingly investigated among individuals living with schizophrenia and other psychotic disorders [9]. Metacognition is associated with fundamental features of schizophrenia, including emotional expression, cognitive abilities and insight, and quality of life [10,11,12]. Research has further shown links between metacognitive abilities and language, specifically that the use of more complex and rich language is related to higher metacognitive abilities [13,14].

Language disturbance can manifest in speech among individuals with schizophrenia in a variety of ways (e.g., derailment, tangentiality, and unintelligible speech) [15,16,17]. Symptoms of language disturbance can hinder interpersonal communication among people with schizophrenia [18], which may further contribute to impaired social interactions and diminished social functioning [19]. Speech disturbance is also associated with other aspects of illness that can contribute to social dysfunction among people with schizophrenia—including cognitive [20] and social cognitive functioning [21]. Collectively, improving our understanding of language disturbance in schizophrenia—and, in particular, how language disturbance may impact social and interpersonal functioning—has the potential to inform and improve interventions that target key functional outcomes for individuals living with schizophrenia.

Research examining language in schizophrenia has increasingly utilized spoken language and narrative methodology to elucidate how linguistic disturbance manifests in naturalistic speech. Narrative methodology provides nuanced information regarding a person’s insight into their lived experience with mental illness [22] as well as the extent of their metacognitive deficits [23]. Narrative development has also been identified as an important factor in psychotherapy for schizophrenia as it can offer perspective into a person’s sense of meaning-making around their life, rather than simple autobiographical facts [24]. Narrative methodology can also provide a more comprehensive view of a person’s level of functioning, possibly expanding beyond the information collected in other self-reported or performance-based assessments within a laboratory setting [25,26]. Finally, this methodological approach offers phenomenological insight directly from the source (i.e., the person with lived experience of the illness), further permitting the sharing of an individual’s perspectives about their own thinking, understanding, and awareness, all of which are related to the phenomenon of metacognition.

Researchers have explored the connection between metacognition and language in schizophrenia using the Metacognitive Assessment Scale-Abbreviated (MAS-A) [27], which was designed to measure metacognitive abilities through spoken life narratives [12,28]. The MAS-A may offer perspective into a person’s metacognitive capacity with more nuance than what could be shown in a more standardized clinical or non-narrative assessment. The MAS-A has also been used to investigate aspects of metacognitive function that may change and relate to other metrics of outcome during the course of treatment among people with schizophrenia [29,30,31]. In studies comparing individuals with schizophrenia and bipolar disorder to non-clinical controls, groups with serious mental illness typically show lower levels of metacognition than controls evidenced by MAS-A scores [32,33,34,35].

Previous studies have explored the associations between MAS-A scores and social functioning among individuals with schizophrenia. For example, James and colleagues [36] found significant associations between metacognition and multiple aspects of rater-based social functioning (i.e., social contact frequency, building and maintaining social relationships), with further evidence that metacognition moderates the relationship between self-appraisal and social functioning. An additional study noted that MAS-A Decentration—an aspect of metacognition related to an individual’s understanding that others have lives and thought processes separate from their own—was positively correlated to the self-reported quality of interpersonal relationships [37]. Additionally, Gagen et al. [28] observed that people with schizophrenia with rater-based moderate to severe impairments in social functioning had lower MAS-A scores than those with good social functioning. Finally, data from Bröcker et al. [38] showed significant associations between all subscales of the MAS-A and a global, interview-rated measure of social functioning. Although this research highlights the significant associations between social functioning and the metacognitive abilities assessed with the MAS-A among individuals with schizophrenia, these existing studies have utilized self-report questionnaires and interview-based methods. Notably, some illness-related factors (e.g., cognitive impairment, diminished insight) may influence the self-reported capacity among people with schizophrenia [39,40], and interview-based methods may miss critical information about the actual nature of social interactions. Thus, the inclusion of performance-based measures which include the direct observation of social behavior has been identified as a key factor in improving the assessment of functional status among people living with psychosis [41] and is important for addressing the limitations of previous work.

In the present study, we performed a secondary data analysis with the following primary aims: (i) assess narrative-derived metacognitive functioning among individuals with schizophrenia and individuals without a psychotic disorder diagnosis, and (ii) examine the associations between metacognition and multifaceted aspects of social functioning among people with schizophrenia. To elicit participant narratives, we used a semi-structured, interview-based approach that permitted a naturalistic, conversation-like flow that may more closely approximate the real-world use of language and its underlying metacognitive components [27,42] (i.e., the Indiana Psychiatric Illness Interview [IPII]; rated using the MAS-A). Social functioning in the present study was assessed via both performance-based assessments and self-reported measures, providing a comprehensive measurement of social functioning. Based on previous research [32,34,35,43], we expected that narrative-derived metacognition scores would be diminished among individuals with schizophrenia relative to individuals without psychosis. We further hypothesized that higher metacognition scores would be associated with better performance on both self-reported and performance-based measures of social functioning among individuals with schizophrenia.

## 2. Methods

### 2.1. Participants

The participants (total *n* = 47) included (i) individuals with schizophrenia and (ii) individuals without a current or historical psychotic disorder diagnosis. The data for this study were drawn from a broader study of language and cognition [44,45]. Participants were excluded if they met any of the following criteria: a history of traumatic brain injury or other organic brain damage, intellectual disability, history of seizures, history of any inhalant use, history of alcohol or substance dependence requiring inpatient detoxification, and/or active substance use disorder. All participants spoke English as their primary language.

2.1.1 Individuals with schizophrenia. Thirty-two individuals meeting the criteria for a DSM-IV-TR [46] diagnosis of schizophrenia (as confirmed via the Schedule for Affective Disorders and Schizophrenia [SADS] [47]) participated in the study. SADS assessments were completed by trained graduate-level researchers and further supervised by a licensed clinical psychologist. Participants with schizophrenia were recruited from a local community mental health clinic.

2.1.2 Individuals without a psychotic disorder diagnosis. An additional fifteen participants who did not have a psychotic disorder diagnosis were recruited to serve as a community control comparison group. Control participants were recruited through flyers in communal areas in Akron, Ohio. All individuals in the control group also underwent the SADS diagnostic interview to rule out any current or historical psychotic symptoms.

### 2.2. Procedure

Participants were assessed in a single session. Written informed consent was obtained from all study participants. Study procedures were approved by the Kent State University Institutional Review Board as well as the Research Merit Board of the community mental health center where individuals in the schizophrenia group were assessed.

### 2.3. Measures

2.3.1 Life-story and Illness Narratives. The Indiana Psychiatric Illness Interview (IPII) [48] is a semi-structured interview in which participants are given an initial prompt to describe the story of one’s life in as much detail as possible. Following the free narrative, participants are asked a standardized set of questions about functioning, interpersonal relationships, and perceptions about the future. This approach allows individuals to provide a spontaneous life narrative that emerges with little to no interviewer interruption or prompting. For the purpose of the present study, we revised a portion of the IPII prompts for the control participants from the wording of “do you have a mental illness?” to “do you believe you have any psychological difficulties?” to make the interview more broadly applicable for administration to this group [45]. The narratives were audio-recorded and later transcribed. Transcription was performed by trained research assistants under the supervision of the senior author (AMM). Each completed transcription was subject to an accuracy check by a second, independent research assistant to verify accuracy prior to data coding and analysis.

2.3.2 Metacognitive Functioning. Metacognitive function was assessed via the Metacognition Assessment Scale—Abbreviated (MAS-A) [27]. The MAS-A is a rating scale designed for the assessment of metacognitive abilities within the context of narratives. It is composed of four domains: Self-reflectivity, Understanding the Other’s Mind, Decentration, and Mastery (Table 1). Higher scores on each domain indicate better metacognitive functioning. The Cronbach’s alpha for the MAS-A subscales in the present study was acceptable (α = 0.73). Although individuals in the control group were not diagnosed with a psychosis spectrum disorder, we completed Mastery (i.e., the ability to cope with psychological difficulties) ratings for both the schizophrenia and control groups in order to (1) provide the same standardized interview prompts and ratings for all participants, and (2) recognize that psychological distress and/or illness are not experienced only by individuals with schizophrenia. The MAS-A ratings were completed by the primary author (MFVK). A subsample of 15 narratives was also independently rated by the senior author (AMM). The intraclass correlation coefficients (ICCs) calculated for each subscale demonstrated acceptable inter-rater reliability (absolute agreement for all subscales > 0.764).

2.3.3 Social Functioning. Social functioning was assessed via both a self-report questionnaire and a performance-based measure of social behavior.

The Social Functioning Questionnaire (SFQ) [49] is an 8-item self-report measure that assesses multiple domains of social functioning and behavior (e.g., functioning at home and work, interpersonal function, and impact of stressors). The participants rate their experience of difficulties on each item with scores ranging from 0 (no problems at all) to 3 (very severe problems). The scores on each item are summed for a total SFQ score, with higher total scores reflecting worse social functioning. The SFQ was developed from the Social Functioning Schedule [50] and has been utilized in clinical research among individuals with schizophrenia and other psychotic disorders [49,51,52].

The Assessment of Interpersonal Problem-Solving Skills (AIPSS) [53] is a performance-based measure that involves the participant watching a series of thirteen videos depicting social interactions. After each video, participants are asked whether a social problem occurred. If the participant identifies a problem, they are asked to describe the problem, describe what they would do or say if they were in the situation, and role-play the solution with the experimenter. Consistent with our previous work using these data [44,54], we derived composite scores that align with the receiving–processing–sending (RPS) model of social problem-solving skills [53,55]. According to the RPS model, successful social problem-solving requires that individuals are able to recognize a social problem as having occurred (receiving), describing a solution to the identified social problem (processing), and enacting a solution to the social problem (sending). The total scores for each subscale (i.e., receiving, processing, and sending) were calculated as the percentages correct out of the items for each participant. Two individuals in the schizophrenia group had missing data for the AIPSS, and one participant’s composite scores were unusable due to only correctly identifying one scene as containing a problem, leaving 29 participants’ data for AIPSS analysis.

### 2.4. Statistical Analyses

Independent samples *t*-tests were utilized to assess the differences in the MAS-A subscale scores between individuals with schizophrenia and individuals without psychosis. We used Bivariate (Pearson’s r) correlations to investigate the associations between MAS-A subscale scores and social functioning variables (SFQ total score and AIPSS subscale scores) among individuals with schizophrenia. All variables were approximately normally distributed.

## 3. Results

The demographic data for all participants appear in Table 2, and the descriptive data for the metacognitive and social functioning variables appear in Table 3. Individuals in the schizophrenia and community control groups did not differ significantly on age, sex, or race. Individuals with schizophrenia did have fewer years of education relative to community controls. The correlations between metacognition and social functioning among individuals with schizophrenia appear in Table 4.

**2.3.1** Metacognitive functioning and group differences. A two-tailed *t*-test revealed that individuals with schizophrenia, relative to individuals without psychosis, had significantly higher scores on the Mastery domain of the MAS-A (t (45) = 3.91, *p* < 0.001; *d* = 1.113). Individuals with schizophrenia did not significantly differ from individuals without psychosis on the Self-Reflectivity, Understanding the Other’s Mind, or Decentration subscales of the MAS-A.

**2.3.2** Metacognition and social functioning among individuals with schizophrenia. Among participants with schizophrenia, the MAS-A Self-Reflectivity scores were negatively correlated with total SFQ scores (r = −0.49, *p* = 0.006). The Understanding of the Other’s Mind scores were positively correlated with AIPSS Sending scores (r = 0.38, *p* = 0.034), and Mastery scores were positively correlated with AIPSS Receiving scores (r = 0.37, *p* = 0.039). No significant correlations were observed between Decentration scores and any of the social functioning scores.

## 4. Discussion

In the present study, we assessed metacognitive functioning from spoken, life-story narratives of people with schizophrenia and community participants with no history of psychosis, and we examined the associations between metacognition and social functioning among individuals with schizophrenia. Broadly, most aspects of narrative-derived metacognitive function were not significantly different between people with schizophrenia and community participants. The one exception was the metacognitive function of Mastery, which was *significantly higher* among people with schizophrenia relative to participants without psychosis. Among individuals with schizophrenia, aspects of metacognitive function were differentially associated with self-reported and performance-based measures of social functioning. Specifically, higher scores on the metacognitive function of Self-Reflectivity were associated with poorer self-reported social functioning, whereas higher scores on the metacognitive functions of Understanding of the Other’s Mind and Mastery were associated with a better performance on aspects of performance-based social functioning (i.e., Sending and Receiving scores from the AIPSS, respectively).

Most aspects of narrative-derived metacognition (i.e., Self-reflectivity, Understanding of the Other’s Mind, and Decentration) were not observed to be significantly different between individuals with schizophrenia and the community controls in this study. This finding is inconsistent with previous research that has found significant differences between groups, where individuals with schizophrenia groups had significantly lower MAS-A scores relative to the control participants [33,35,56]. Notably, previous research has found that when individuals with schizophrenia are grouped according to their clinical and functional presentation, those in higher-functioning subgroups evidence higher scores across MAS-A subscales [28]. Another possibility is selection bias, as this study involved individuals who choose to participate in life-story narrative research. It is possible that individuals who are less comfortable or interested in sharing their life-story narratives as a part of a research study may have received different MAS-A scores or evidenced different patterns of associations between metacognitive and social functioning. Finally, it is important to acknowledge that the MAS-A scores observed in our community control comparison sample were lower than the scores observed in other published studies with non-psychosis comparison groups [33,35,38,56,57]. Psychological distress—and the influence that this distress can have on metacognitive functioning—is a broad construct that crosses diagnostic and clinical versus non-clinical boundaries. In this study, individuals remained eligible for the community comparison group if they had other, non-psychotic forms of psychopathology. Although we are unable to confirm specific contributions to the lower MAS-A scores among the community controls observed in the present study, this participant selection approach may have influenced our results by minimizing between-group differences in metacognition.

Also unexpectedly, we found the metacognitive aspect of Mastery to be higher in the schizophrenia group relative to the community controls. One possible interpretation of this finding is that individuals with serious mental illness, relative to individuals without psychosis, may more often face a serious disruption to their functioning and daily lives secondary to the onset of psychosis. This experience may, for some individuals, necessitate the development of coping strategies and illness management skills that manifest as higher levels of illness insight and mastery in life-story narratives. Stated differently, the use of life-story narrative methodology enhanced with specific questioning about the influence of mental illness on one’s life and experiences may have allowed individuals in the schizophrenia group to demonstrate their awareness of their experience with psychological problems, their ability to respond to related challenges, and/or to otherwise express themes of resiliency that would not have been apparent without a narrative approach to measurement. Of note, participants in the schizophrenia group were recruited from a community mental healthcare center while community controls were recruited via flyers placed in non-clinical settings (i.e., local churches, community centers, and throughout the city). Although involvement in treatment was not a requirement for participation, and nor was it assessed as part of the current study, this approach to recruitment likely contributed to a greater representation of individuals with either current or historical psychiatric treatment among the schizophrenia group. Should this be accurate, a lack of being exposed to mental health treatment could also influence the content generated during the life-story narrative, as community control participants may have had fewer or different opportunities to speak about or process their psychological difficulties. Similarly, the individuals without psychosis may not have identified particular mental health problems in their experiences that could have led to further discussion on the content related to Mastery in their life-story narratives. With regard to previous research, although the Mastery scores observed in our schizophrenia participants were higher than those documented in some studies [33,35,38,56,57], others have noted higher MAS-A Mastery scores among people with schizophrenia that approximate and exceed the scores in the present study [28,34,43,58].

There were differential associations between aspects of metacognition and social functioning measures, some of which aligned with study hypotheses. More specifically, better metacognitive ability was associated with poorer self-reported functioning, but better scores on aspects of performance-based functioning. This finding underscores the nuance of social functioning, as differential associations emerge when social functioning is measured in multiple ways and not in a simple composite manner. This also reflects a double-edged sword of insight and awareness [59], as being more self-reflective may in some cases make a person more aware of difficulties and thus, more likely to report their presence and severity relative to someone with less reflectivity. With regard to the performance-based measure of social functioning (AIPSS), we first observed that the Understanding of the Other’s Mind was related specifically to Sending scores (i.e., the ability to demonstrate how one would enact a solution to a social problem they have identified). This finding provides interesting evidence that how individuals with schizophrenia describe their awareness of the internal states of others during the course of their life-story narratives is significantly and positively correlated to how well they were able to demonstrate the execution of interpersonal social problem-solving skills. We further found that Mastery was positively correlated with Receiving scores (i.e., the ability to accurately recognize that a social problem had occurred). This finding indicates that the metacognitive ability of coping with situations of distress related to the accurate identification of problems in social circumstances. One interpretation of this pattern of results is that individuals with schizophrenia who show a higher metacognitive aptitude in the awareness of their own thought processes and those of others, as well as the ability to use this knowledge to better cope with sources of distress, have more facility to identify and cope with adverse social situations.

In summary, using multiple methods of social functioning measurement in this study has provided information on both one’s self-perception and one’s actual ability to describe and demonstrate social problem-solving skills, thus allowing more specific patterns of associations between metacognition and social functioning to emerge. These findings also have clinical implications, as they highlight the importance of considering narrative and narrative-derived indices of functioning among individuals with mental illness. Particularly, the association between metacognition and self-reported functioning and performance-based functioning scores demonstrates how incorporating a narrative with multiple social functioning measurements can provide a more nuanced understanding of a person’s level of metacognitive and social functioning. This could provide clinicians with further insight into patients’ metacognitive and social functioning, which could then better inform treatment plans and observed progress. Collectively, our results suggest that aspects of metacognitive functioning embedded within life-story narratives relate to insight into and the performance of social functions, which could inform therapeutic targets (e.g., using Social Skills Training [60]; and/or Metacognitive Insight and Reflection Therapy [61]) and/or monitoring mechanisms of change (e.g., using the MAS-A, SFQ, and AIPSS) during the course of treatment.

### 4.1. Strengths

The strengths of this study include our use of life-story narratives to assess metacognitive functioning, our inclusion of individuals without psychosis as a comparison group, and the use of both self-report and performance-based measurements of social functioning. Narrative prompts can allow metacognitive functioning to emerge through one’s life story, offering the potential to understand the nuance of the real-world implementation of metacognition that may not be assessed in other standard, lab-based tasks. The use of both self-report and performance-based social functioning assessments provided a more comprehensive assessment of social functioning and how different aspects of the social process may relate to metacognitive function among people with schizophrenia. Including community controls who also completed the IPII allows for further recognition that psychological difficulties span the continuum of clinical and community individuals.

### 4.2. Limitations

Our study also has important limitations. First, we do not have specific information on the possible presence or absence of psychiatric comorbidities among study participants. Individuals in either the schizophrenia group or the control group were eligible for participation if they had other comorbid psychiatric diagnoses, as only the presence of an active substance use disorder or a history of psychosis were assessed and ruled-out for the purposes of determining the eligibility criteria. Thus, the presence of comorbidities and their possible influence on our pattern of results remain unknown. Relatedly, medication data are not available for participants in the present study and the possible influence of psychiatric medications is unknown. Next, our assessment of metacognition was limited to the MAS-A in the present study. Using different assessments of metacognition could provide a more comprehensive evaluation of participants’ metacognitive ability and its contributions to social functioning. Notably, this study was conducted before a control version of the IPII [33] was available, and instead we modified the interview to be appropriate for our comparison group. The wording of the question “Do you believe you have a mental illness?” was edited into “Do you believe you have any psychological problems?” for the control population. Whether our pattern of results would be replicated when the updated version of the IPII for community control participants is utilized is not known. Third, multiple comparisons were conducted without statistical correction due to the small within-group sample sizes and thus, the results should be interpreted cautiously. Next, we had uneven sample sizes for the community comparison and schizophrenia groups. We cannot rule out the possibility that our use of a smaller control group may have impacted our results, particularly as some aspects of metacognitive scores in this group were lower/inconsistent with previous studies [33,56]. Relatedly, the small sample size in the present study requires that our current results be considered as preliminary and thus interpreted with appropriate caution.

### 4.3. Future Directions

Our findings have several implications for future research. First, additional studies examining the associations and comparisons between MAS-A scores and social functioning among youth and young adults in the early stages of psychosis are warranted. In particular, larger studies with specific planned and/or pre-registered analyses related to language-derived metacognitive function may provide a replication and refinement of our current pattern of results. Our participants were in a more chronic and established phase of illness, and thus, examining these same variables in first-episode psychosis or with youth at clinical high-risk for developing a psychotic disorder could provide more information on the influence of the illness phase on metacognition and its association with social functioning. Building upon our limitations, future studies that utilize the control version of the IPII [33] could potentially provide additional comparisons to our results. Our findings also provide additional support for the relevance of narrative and metacognition-focused forms of psychotherapy for individuals with schizophrenia [29], as these approaches allow room for narrative expression that can provide the clinician with more information about metacognitive functioning, possibly rendering the opportunity to better adapt treatment and enhance its effectiveness for individuals with schizophrenia. For example, our results suggest that the narrative method may allow aspects of metacognitive functioning to emerge naturally while providing insight on an individual level, which may be particularly important in understanding social outcomes as these aspects were related in our results. Relatedly, additional studies that measure and integrate aspects of the lived experience of psychosis—via a life-story narrative or other methodologies—will be important in furthering this area of research. Finally, future studies assessing the relationship of metacognitive functioning to psychopathology may benefit from a consideration of additional comparison groups with different types of presenting concerns. These could include individuals diagnosed with depression, anxiety, or trauma, to better understand how metacognition is impacted in schizophrenia versus other forms of mental illness.

## Figures and Tables

**Table 1:** MAS-A subscale descriptions.

Subscale	Description
Self-Reflectivity	Evaluates the extent to which individuals convey an understanding of their own mental and emotional processes
Understanding Other’s Mind	Evaluates one’s ability to understand and consider the mental processes of other individuals
Decentration	Assesses the extent to which individuals convey an understanding that the lives of others are not centered around one’s self
Mastery	Evaluates one’s understanding of their own mental illness and/or psychological difficulties and the ability to effectively manage or control these problems

From Lysaker, Buck, and Hamm [27].

**Table 2:** Participant characteristics.

		Schizophrenia Group	Community Controls	Group Difference Statistics
N		32	15	
Mean Age (SD)		41.66 (7.75)	38.87 (9.64)	t (45) = −1.063, *p* = 0.293
Sex (%)				*χ*² = 0.170, df = 1, *p* = 0.680
Male		17 (53)	7 (46)	
Female		15 (47)	8 (54)	
Race (%)				*χ*² = 1.047, df = 1, *p* = 0.306
Black		20 (63)	8 (54)	
Caucasian		12 (37)	7 (46)	
Mean Years of Education (SD)		11.89 (1.31)	13.67 (2.38)	t (45) = 3.311, *p* = 0.002 *

* *p* < 0.05.

**Table 3:** Metacognition indices in life-story narratives.

	Schizophrenia Group	Community Controls	Group Different Statistics
Mean Word Count	1720.03 (880.70)	2217.67 (1717.88)	t (45) = 1.059, *p* = 0.304
For Narrative (SD)			
MAS-A Subscales (SD)			
*Self-Reflectivity*	3.641 (0.651)	3.667 (0.919)	t (45) = 0.112, *p* = 0.456
*Understanding Other’s Mind*	2.203 (1.436)	2.200 (1.521)	t (45) = 0.007, *p* = 0.497
*Decentration*	0.375 (0.524)	0.600 (0.431)	t (45) = 1.448, *p* = 0.077
*Mastery*	4.453 (1.531)	2.200 (2.389)	t (45) = 3.910, *p* < 0.001 **

** *p* < 0.001.

**Table 4:** Correlations between metacognition and social functioning variables among individuals with schizophrenia.

	AIPSS Receiving	AIPSS Processing	AIPSS Sending	SFQ
Self-Reflectivity	0.043	−0.094	−0.163	−0.486 **
Understanding Other’s Mind	0.237	0.243	0.382 *	0.047
Decentration	0.044	0.037	0.151	0.218
Mastery	0.373 *	0.161	0.291	−0.121

AIPSS = Assessment of Interpersonal Problem-Solving Skills. SFQ = Social Functioning Question. * *p* < 0.05. ** *p* < 0.001.

## Data Availability

Due to the nature of this study, participants did not consent for their data to be shared publicly. Therefore, the data that support the findings of this study are not publicly available due to privacy or ethical restrictions.

## References

[1] Charlson F.J., Ferrari A.J., Santomauro D.F., Diminic S., Stockings E., Scott J.G., McGrath J.J., Whiteford H.A. (2018). Global Epidemiology and Burden of Schizophrenia: Findings from the Global Burden of Disease Study 2016. Schizophr. Bull..

[2] Breitborde N.J.K., López S.R., Chang C., Kopelowicz A., Zarate R. (2009). Emotional Over-Involvement Can Be Deleterious for Caregivers’ Health: Mexican Americans Caring for a Relative with Schizophrenia. Soc. Psychiatry Psychiatr. Epidemiol..

[3] Cloutier M., Aigbogun M.S., Guerin A., Nitulescu R., Ramanakumar A.V., Kamat S.A., DeLucia M., Duffy R., Legacy S.N., Henderson C. (2016). The Economic Burden of Schizophrenia in the United States in 2013. J. Clin. Psychiatry.

[4] Magliano L., Fiorillo A., De Rosa C., Malangone C., Maj M. (2005). Family Burden in Long-Term Diseases: A Comparative Study in Schizophrenia vs. Physical Disorders. Soc. Sci. Med..

[5] Pinna F., Deriu L., Lepori T., Maccioni R., Milia P., Sarritzu E., Tusconi M., Carpiniello B. (2013). Is It True Remission? A Study of Remitted Patients Affected by Schizophrenia and Schizoaffective Disorders. Psychiatry Res..

[6] Karow A., Moritz S., Lambert M., Schöttle D., Naber D. (2012). Remitted but Still Impaired? Symptomatic versus Functional Remission in Patients with Schizophrenia. Eur. Psychiatry.

[7] Turner P.R., Saeteurn E.R. (2022). Social Satisfaction and Living Alone: Predictors of Self-Perception of Mental Health Improvement after Psychosis. Schizophr. Bull. Open.

[8] Lysaker P.H., McCormick B.P., Snethen G., Buck K.D., Hamm J.A., Grant M., Nicolò G., Dimaggio G. (2011). Metacognition and Social Function in Schizophrenia: Associations of Mastery with Functional Skills Competence. Schizophr. Res..

[9] Martiadis V., Pessina E., Raffone F., Iniziato V., Martini A., Scognamiglio P. (2023). Metacognition in Schizophrenia: A Practical Overview of Psychometric Metacognition Assessment Tools for Researchers and Clinicians. Front. Psychiatry.

[10] García-Mieres H., Lundin N.B., Minor K.S., Dimaggio G., Popolo R., Cheli S., Lysaker P.H. (2020). A Cognitive Model of Diminished Expression in Schizophrenia: The Interface of Metacognition, Cognitive Symptoms and Language Disturbances. J. Psychiatr. Res..

[11] Mutlu E., Abaoğlu H., Barışkın E., Gürel C., Ertuğrul A., Yazıcı M.K., Akı E., Yağcıoğlu A.E.A. (2021). The Cognitive Aspect of Formal Thought Disorder and Its Relationship with Global Social Functioning and the Quality of Life in Schizophrenia. Soc. Psychiatry Psychiatr. Epidemiol..

[12] Wright A.C., Lysaker P.H., Fowler D., Greenwood K. (2023). Clinical Insight in First Episode Psychosis: The Role of Metacognition. J. Ment. Health.

[13] Buck B., Minor K.S., Lysaker P.H. (2015). Differential Lexical Correlates of Social Cognition and Metacognition in Schizophrenia; A Study of Spontaneously-Generated Life Narratives. Compr. Psychiatry.

[14] Lundin N.B., Hochheiser J., Minor K.S., Hetrick W.P., Lysaker P.H. (2020). Piecing Together Fragments: Linguistic Cohesion Mediates the Relationship between Executive Function and Metacognition in Schizophrenia. Schizophr. Res..

[15] Kuperberg G.R. (2010). Language in Schizophrenia Part 1: An Introduction. Linguist. Lang. Compass.

[16] Rodriguez-Ferrera S., McCarthy R.A., McKenna P.J. (2001). Language in Schizophrenia and Its Relationship to Formal Thought Disorder. Psychol. Med..

[17] Sevilla G., Rosselló J., Salvador R., Sarró S., López-Araquistain L., Pomarol-Clotet E., Hinzen W. (2018). Deficits in Nominal Reference Identify Thought Disordered Speech in a Narrative Production Task. PLoS ONE.

[18] Pienkos E., Sass L.A. (2016). Expressions of Alienation: Language and Interpersonal Experience in Schizophrenia. J. Psychopathol..

[19] Roche E., Segurado R., Renwick L., McClenaghan A., Sexton S., Frawley T., Chan C.K., Bonar M., Clarke M. (2015). Language Disturbance and Functioning in First Episode Psychosis. Psychiatry Res..

[20] Docherty N.M., Strauss M.E., Dinzeo T.J., St-Hilaire A. (2006). The Cognitive Origins of Specific Types of Schizophrenic Speech Disturbances. Am. J. Psychiatry.

[21] Docherty N.M., McCleery A., Divilbiss M., Schumann E.B., Moe A., Shakeel M.K. (2013). Effects of Social Cognitive Impairment on Speech Disorder in Schizophrenia. Schizophr. Bull..

[22] Roe D., Hasson-Ohayon I., Kravetz S., Yanos P.T., Lysaker P.H. (2008). Call It a Monster for Lack of Anything Else: Narrative Insight in Psychosis. J. Nerv. Ment. Dis..

[23] Lysaker P.H., Dimaggio G., Buck K.D., Carcione A., Nicolò G. (2007). Metacognition within Narratives of Schizophrenia: Associations with Multiple Domains of Neurocognition. Schizophr. Res..

[24] Hamm J.A., Leonhardt B.L. (2016). The Role of Interpersonal Connection, Personal Narrative, and Metacognition in Integrative Psychotherapy for Schizophrenia: A Case Report. J. Clin. Psychol..

[25] Lysaker P.H., Erickson M., Buck K.D., Procacci M., Nicolò G., Dimaggio G. (2010). Metacognition in Schizophrenia Spectrum Disorders: Methods of Assessment and Associations with Neurocognition and Function. Eur. J. Psychiatry.

[26] Lysaker P.H., Hillis J., Leonhardt B.L., Kukla M., Buck K.D. (2014). Metacognition in Schizophrenia Spectrum Disorders: Methods of Assessment and Associations with Neurocognition, Symptoms, Cognitive Style and Function. Isr. J. Psychiatry Relat. Sci..

[27] Lysaker P., Buck K., Hamm J.A. (2011). Metacognition Assessment Scale: A Brief Overview and Coding Manual for the Abbreviated Version.

[28] Gagen E.C., Zalzala A.B., Hochheiser J., Martin A.S., Lysaker P.H. (2019). Metacognitive Deficits and Social Functioning in Schizophrenia across Symptom Profiles: A Latent Class Analysis. J. Exp. Psychopathol..

[29] De Jong S., Van Donkersgoed R.J.M., Timmerman M.E., Aan Het Rot M., Wunderink L., Arends J., Van Der Gaag M., Aleman A., Lysaker P.H., Pijnenborg G.H.M. (2019). Metacognitive Reflection and Insight Therapy (MERIT) for Patients with Schizophrenia. Psychol. Med..

[30] Lavi-Rotenberg A., Roe D., Igra L., Hasson-Ohayon I. (2023). Re-Owning Motherhood in Metacognitive Reflection and Insight Therapy (MERIT) with A Woman Diagnosed with Schizophrenia: Lessons from A Case Study. J. Contemp. Psychother..

[31] Inchausti F., García-Mieres H., García-Poveda N.V., Fonseca–Pedrero E., MacBeth A., Popolo R., Dimaggio G. (2023). Recovery-Focused Metacognitive Interpersonal Therapy (MIT) for Adolescents with First-Episode Psychosis. J. Contemp. Psychother..

[32] Aydin O., Balikci K., Tas C., Aydin P.U., Danaci A.E., Brüne M., Lysaker P.H. (2016). The Developmental Origins of Metacognitive Deficits in Schizophrenia. Psychiatry Res..

[33] Davis B.J., Bonfils K.A., Zalzala A., Lysaker P.H., Minor K.S. (2022). Meaning-Making Processes across the Lifespan: An Investigation of the Developmental Course of Metacognitive Capacity. Schizophr. Res..

[34] Mediavilla R., López-Arroyo M., Gómez-Arnau J., Wiesepape C., Lysaker P.H., Lahera G. (2021). Autobiographical Memory in Schizophrenia: The Role of Metacognition. Compr. Psychiatry.

[35] Popolo R., Smith E., Lysaker P.H., Lestingi K., Cavallo F., Melchiorre L., Santone C., Dimaggio G. (2017). Metacognitive Profiles in Schizophrenia and Bipolar Disorder: Comparisons with Healthy Controls and Correlations with Negative Symptoms. Psychiatry Res..

[36] James A.V., Hasson-Ohayon I., Vohs J., Minor K.S., Leonhardt B.L., Buck K.D., George S., Lysaker P.H. (2016). Metacognition Moderates the Relationship between Dysfunctional Self-Appraisal and Social Functioning in Prolonged Schizophrenia Independent of Psychopathology. Compr. Psychiatry.

[37] Fischer M.W., Dimaggio G., Hochheiser J., Vohs J.L., Phalen P., Lysaker P.H. (2020). Metacognitive Capacity Is Related to Self-Reported Social Functioning and May Moderate the Effects of Symptoms on Interpersonal Behavior. J. Nerv. Ment. Dis..

[38] Bröcker A.L., Bayer S., Stuke F., Giemsa P., Heinz A., Bermpohl F., Lysaker P.H., Montag C. (2017). The Metacognition Assessment Scale (MAS-A): Results of a Pilot Study Applying a German Translation to Individuals with Schizophrenia Spectrum Disorders. Psychol. Psychother. Theory Res. Pract..

[39] Atkinson M., Zibin S., Chuang H. (1997). Characterizing Quality of Life among Patients with Chronic Mental Illness: A Critical Examination of the Self-Report Methodology. Am. J. Psychiatry.

[40] Durand D., Strassnig M., Sabbag S., Gould F., Twamley E.W., Patterson T.L., Harvey P.D. (2015). Factors Influencing Self-Assessment of Cognition and Functioning in Schizophrenia: Implications for Treatment Studies. Eur. Neuropsychopharmacol..

[41] Bowie C.R., Twamley E.W., Anderson H., Halpern B., Patterson T.L., Harvey P.D. (2007). Self-Assessment of Functional Status in Schizophrenia. J. Psychiatr. Res..

[42] Lysaker P.H., Buck K.D. (2009). Metacognition in Schizophrenia Spectrum Disorders: Methods of Assessing Metacognition within Narrative and Links with Neurocognition. Ital. J. Psychopathol..

[43] Lysaker P.H., Irarrázaval L., Gagen E.C., Armijo I., Ballerini M., Mancini M., Stanghellini G. (2018). Metacognition in Schizophrenia Disorders: Comparisons with Community Controls and Bipolar Disorder: Replication with a Spanish Language Chilean Sample. Psychiatry Res..

[44] Moe A.M., Breitborde N.J.K., Bourassa K.J., Gallagher C.J., Shakeel M.K., Docherty N.M. (2018). Schizophrenia, Narrative, and Neurocognition: The Utility of Life-Stories in Understanding Social Problem-Solving Skills. Psychiatr. Rehabil. J..

[45] Moe A.M., Breitborde N.J.K., Shakeel M.K., Gallagher C.J., Docherty N.M. (2016). Idea Density in the Life-Stories of People with Schizophrenia: Associations with Narrative Qualities and Psychiatric Symptoms. Schizophr. Res..

[46] American Psychiatric Association (2000). Diagnostic and Statistical Manual of Mental Disorders.

[47] Endicott J., Spitzer R.L. (1978). A Diagnostic Interview: The Schedule for Affective Disorders and Schizophrenia. Arch. Gen. Psychiatry.

[48] Lysaker P.H., Clements C.A., Plascak-Hallberg C.D., Knipscheer S.J., Wright D.E. (2002). Insight and Personal Narratives of Illness in Schizophrenia. Psychiatry.

[49] Tyrer P., Nur U., Crawford M., Karlsen S., McLean C., Rao B., Johnson T. (2005). The Social Functioning Questionnaire: A Rapid and Robust Measure of Perceived Functioning. Int. J. Soc. Psychiatry.

[50] Remington M., Tyrer P. (1979). The Social Functioning Schedule—A Brief Semi-Structured Interview. Soc. Psychiatry.

[51] Fitah I., Chakit M., El Kadiri M., Brikat S., El Hessni A., Mesfioui A. (2023). The Evaluation of the Social Functioning of Schizophrenia Patients Followed up in the Health Center My El Hassan of Kenitra, Morocco. Egypt. J. Neurol. Psychiatry Neurosurg..

[52] Shoham N., Lewis G., Hayes J., McManus S., Kiani R., Brugha T., Bebbington P., Cooper C. (2020). Psychotic Symptoms and Sensory Impairment: Findings from the 2014 Adult Psychiatric Morbidity Survey. Schizophr Res.

[53] Donahoe C.P., Carter M.J., Bloem W.D., Hirsch G.L., Laasi N., Wallace C.J. (1990). Assessment of Interpersonal Problem-Solving Skills. Psychiatry.

[54] Lundin N.B., Cowan H.R., Singh D.K., Moe A.M. (2023). Lower Cohesion and Altered First-Person Pronoun Usage in the Spoken Life Narratives of Individuals with Schizophrenia. Schizophr. Res..

[55] Wallace C.J., Nelson C.J., Liberman R.P., Aitchison R.A., Lukoff D., Elder J.P., Ferris C. (1980). A Review and Critique of Social Skills Training with Schizophrenic Patients. Schizophr. Bull..

[56] Hasson-Ohayon I., Avidan-Msika M., Mashiach-Eizenberg M., Kravetz S., Rozencwaig S., Shalev H., Lysaker P.H. (2015). Metacognitive and Social Cognition Approaches to Understanding the Impact of Schizophrenia on Social Quality of Life. Schizophr. Res..

[57] Ladegaard N., Larsen E.R., Videbech P., Lysaker P.H. (2014). Higher-Order Social Cognition in First-Episode Major Depression. Psychiatry Res..

[58] Davis B.J., Firmin R.L., Lysaker P.H., Salyers M.P., McGrew J., Minor K.S. (2020). An Investigation of Metacognition in Schizotypy: Evidence of Linkage with Negative Traits. Transl. Issues Psychol. Sci..

[59] Vrbova K., Prasko J., Ociskova M., Latalova K., Holubova M., Grambal A., Slepecky M. (2017). Insight in Schizophrenia—A Double-Edged Sword?. Neuroendocrinol. Lett..

[60] Bellack A.S., Mueser K.T., Gingerich S., Agresta J. Social skills training for schizophrenia: A step-by-step guide. Guilford Publications..

[61] Lysaker P.H., Klion R.E. Recovery, Meaning-Making, and Severe Mental Illness: A Comprehensive Guide to Metacognitive Reflection and Insight Therapy.

